# Maresins: The Mainstay in Periodontal Resolution

**DOI:** 10.7759/cureus.21742

**Published:** 2022-01-30

**Authors:** Hima Bindu Gireddy, Haripriya Rajaram, Rekha R Koduganti, Manasa Ambati, Anilkumar R, Tata Sai Lakshmi Harika

**Affiliations:** 1 Department of Periodontics, Panineeya Mahavidyalaya Institute of Dental Sciences and Research Centre, Hyderabad, IND

**Keywords:** periodontitis, macrophage, maresins, inflammation, resolution

## Abstract

Not much was known about the resolution of inflammation until the recent past when significant breakthroughs led to the unveiling of the exact mechanism of this. It is now known that the resolution of inflammation involves specific mediators of resolution such as lipoxins, protectins, resolvins, and maresins, making it an active process. Of these mediators, maresins are the latest discovery. Maresins are macrophage-derived mediators that are involved in the resolution of inflammation. Various studies on what maresins do to resolve periodontitis are ongoing. Reportedly, maresins help in periodontal regeneration and wound healing. Having known the numerous roles of these mediators, our current focus is shifting from anti-inflammatory pharmacotherapy to resolution pharmacotherapy.

## Introduction and background

Living tissue reacts to injury from any agent via a process called inflammation. Normally, inflammation eliminates pathogenic microbes and promotes tissue homeostasis. Nevertheless, a disarrayed or exaggerated response can become pathological and cause unwanted tissue damage [1]. Chronic periodontitis, which is an infectious disease, results in inflammation within the surrounding dental tissues and leads to a reduction in attachment and bone loss. The chief players in the destruction of the periodontium can be broadly divided into two groups: those derived from the subgingival microbiota and those derived from the host immune-inflammatory response [2]. Of the two, it is now clear that most of the periodontal breakdown results from the host’s inflammatory processes. Thus, this inflammation must be resolved to inhibit the progression of periodontitis.

Resolution of inflammation begins with the cellular mechanism, i.e., after entering the tissues, neutrophils interact with the arachidonic acid metabolites, which are converted to lipoxins. This initiates the termination sequence. This ends neutrophil recruitment and apoptosis, i.e., programmed death occurs. Omega-3-derived protectins, resolvins, and maresins induce apoptosis of the neutrophils, thus controlling their infiltration. Consequently, macrophages phagocytize apoptotic neutrophils, leading to clearance of neutrophils and the release of reparative and anti-inflammatory moieties like transforming growth factor-ß1. The sequence terminates with the exit of macrophages via the lymphatics [3].

## Review

Synthesis of specialized proresolving molecules

Specialized proresolving molecules (SPM) are a group of fatty acids. They are an essential part of the cell membrane and act as metabolic fuel for mammalian tissue. Besides these functions, they also work as signaling molecules, thereby having an impact on human health. Omega-6 fatty acids (e.g., linoleic acid [LA]) and omega-3 fatty acids (e.g., a-linolenic acid [ALA]) are essential fatty acids that cannot be biosynthesized by humans or other mammals. After a sequence of elongation and desaturation reactions, LA and ALA transform into their higher unsaturated forms: arachidonic acid from LA and eicosapentaenoic acid (EPA) and docosahexaenoic acid (DHA) from ALA. These further undergo biochemical processing and are transformed into molecules that influence metabolism directly [4].

**Figure 1:**
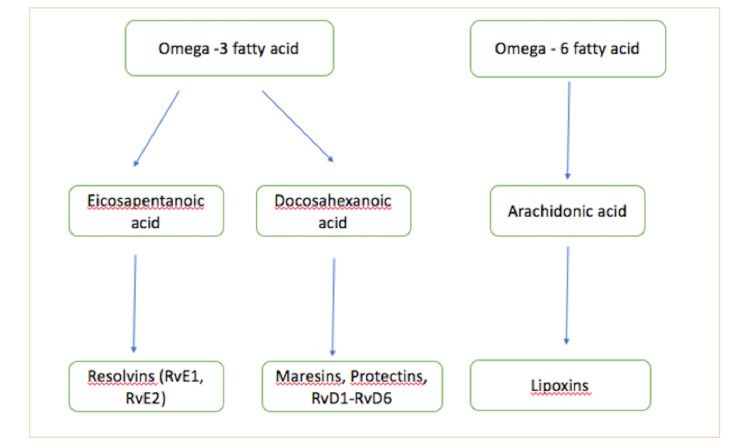
Synthesis of specialized proresolving mediators

The proresolving lipid mediators include lipoxins, protectins, resolvins, and maresins (Figure 1). Lipoxins and aspirin-triggered lipoxins, generated from arachidonic acid, decrease inflammation and assist in resolution. On the contrary, the omega-3 fatty acid is the source of resolvins, maresins, and protectins.

Maresins

"Macrophage mediator in resolving inflammation," maresin-1 (7R,14S-dihydroxydocosa-4Z,8E,10E,12E,16Z,19Z-hexaenoic acid) resolves acute inflammation [5], thus helping in organ protection. It was discovered by Serhan et al. in the year 2009 [6]. Maresins form the third-largest family of SPMs derived from DHA20. The biosynthesis of maresins occurs mainly in M2 macrophages and is initiated by reactions involving human macrophage 12-lipoxygenase (12-LOX) [7].

The generation of maresins begins with the identification of a novel pathway that converts DHA into a 14-hydroxy DHA intermediate. This requires the insertion of an oxygen atom into DHA at the fourteenth carbon atom. This gives rise to 14S-HpDHA, which is further modified into 13S,14S-epoxide maresin, accompanied by a conversion performed by different enzymes to form maresins [4]. Of these, maresin 1 was the first discovered [8].

**Figure 2:**
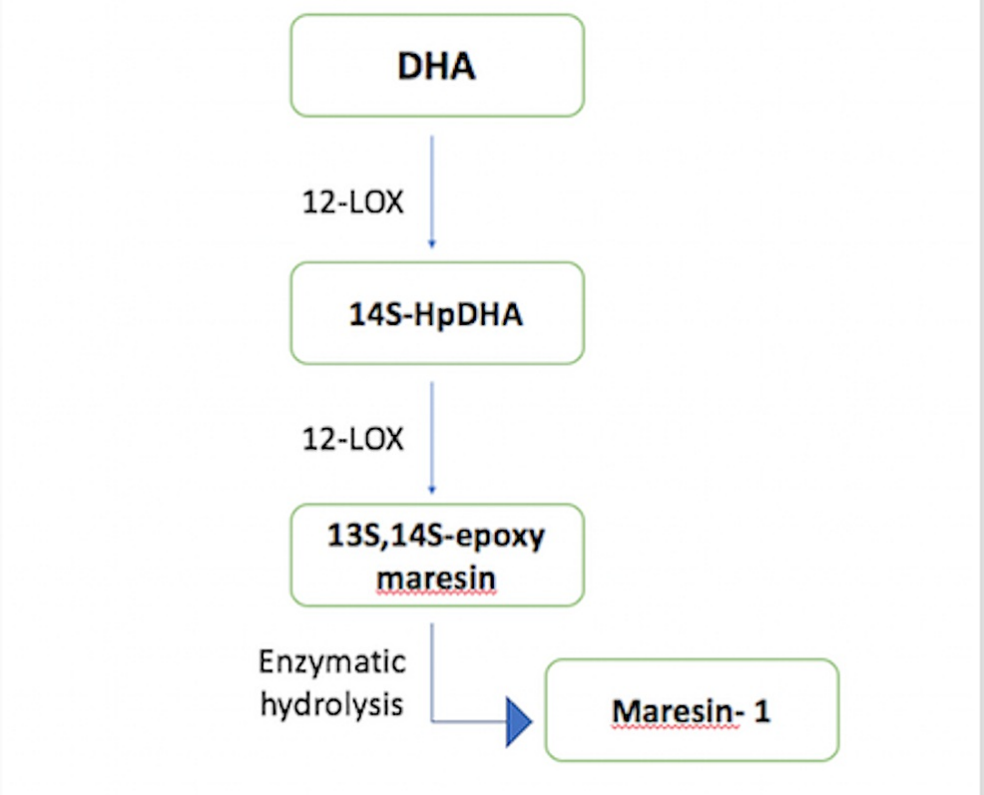
Synthesis of maresins

Other maresin-like oxygenated compounds are built from 22:5(n-3) and 22:5(n-6) fatty acids [9]. These have anti-inflammatory properties. Some dioxygenated metabolites similar to maresins, such as 14S, 21S-dihydroxy-docosa- 4Z,7Z,10Z,12E,16Z,19Z-hexaenoic acid and its epimers are generated via repeated oxidation using 12-LOX and the family of CYP450 enzymes. They are present in macrophages and are found to assist in wound healing [9] (Figure 2).

Receptors of maresins

Two autonomous examinations have uncovered the receptors for maresin. Researchers stated that retinoic acid-related orphan receptor a (RORα) is a maresin-1 receptor producing non-alcoholic steatohepatitis (NASH) [10]. Some other authors found, after screening around 200 G protein-coupled receptors, that maresin-1 is a ligand for human leucine-rich repeat-containing G protein-coupled receptor 6 (LGR6), which is expressed in phagocytes [5,11].

RORα as a Maresin-1 Receptor

It is considered a therapeutic strategy in various chronic inflammatory diseases to switch from the M1 proinflammatory macrophage to the M2 anti-inflammatory macrophage. Han et al. claimed that DHA metabolites may stimulate M2 polarization in the liver through RORα, and the SPMs derived from DHA were tested to determine if they act as RORα ligands [10,12]. Of the investigated SPMs, maresin-1 was discovered to be a specific endogenous ligand of RORα [10]. Maresin-1 induced expression of RORα in liver macrophages caused the generation of 12-LOX, an important enzyme in maresin-1 biosynthesis, and thus further enhanced the level of maresin-1. According to the authors, the liver might have an autoregulatory circuit involving maresin-1/RORa/12-LOX [10]. Macrophages of the liver express higher amounts of 12-LOX than do macrophages of other types [10], showing that the maresin-1/RORa/12-LOX autoregulatory circuit in the liver is a very unique mechanism.

LGR6 as a Receptor for Maresin-1

G-protein-coupled receptors have been found to be target molecules for SPMs, such as lipoxin A4 receptor/formyl peptide receptor 2 (ALX/FPR2) for lipoxin A4, GPR32 for resolvin D1, ChemR23 for resolvin E1, GPR18 for resolvin D2, and GPR37 for protectin D1. Leucine-rich repeat-containing G-protein-coupled receptor 6 (LGR6) has been recognized as a receptor of the SPM maresin-1 [5,11,12]. Chiang et al. showed that maresin-1 stimulated efferocytosis and phagocytosis in an LGR6-dependent manner and that Gs/cAMP signaling influences LGR6 action [11]. This finding provides the missing link in the mystery of maresin-1 pharmacology.

The receptors for maresins act very quickly, and the maresin-1 receptor is responsible for phagocytosis and efferocytosis [7,10]. It was found that maresin-1 inhibited the chemotaxis of neutrophils, suggesting that it may have a polymorphonuclear leukocyte (PMN) recruitment limiting effect, which is another key proresolving mediator action besides efferocytosis/phagocytosis [7,11]. Chiang et al. elaborately studied maresins and observed that maresin-1 carboxymethylester exhibited equal activity on LGR6, but that cysteinyl-containing maresin did not [11]. Maresin 1 was noted to be specific for LGR6. R-spondin-2 and other SPMs were also tested, but no activation was observed for these [11].

Biological actions and possible mechanisms of maresins

SPMs inhibit inflammation-induced tissue damage by downregulating mediators of inflammation, upregulating anti-inflammatory mediators, and limiting tissue infiltration by neutrophils [13]. Maresins as a category of effective new anti-inflammatory mediators have a positive effect on reducing inflammation, oxidative stress, and immune diseases. Current research shows that the cell actions of maresins mainly promote the polarization and phagocytosis of M2 macrophages [14], inhibit the infiltration of neutrophils [15], and induce Treg generation [16]. Maresin 1 deters the differentiation of naive T cells into T-helper 1 (TH1) and T-helper 17 (TH17), and supports differentiation into Treg [17]. In various sites of inflammation, maresins hinder the expression of proinflammatory cytokines such as interleukin 6 (IL-6), tumor necrosis factor alpha (TNF-α), and interleukin 1 beta (IL-lß) by inhibiting the (TLR4/MAPK/NFkB) signaling pathway [18]. Also, maresins can prevent the expression of transforming growth factor beta-1 (TGF-ß1), thereby inhibiting the phenotypic transformation of fibroblasts by inhibiting the ERK/Smad signaling pathway. Maresins upregulate the expression of detoxification and oxidoreductase enzymes by activating the Nrf-2 signaling pathway while reducing reactive oxygen species generation by inhibiting the NFkB signaling pathway.

Maresins and their role in aggressive periodontitis

It has been found that the biosynthesis of MaR1 is dysregulated in cells of localized aggressive periodontitis (LAP) and that the ability of the phagocytes to phagocytize and kill the periodontal pathogens, Porphyromonas gingivalis and Aggregatibacter actinomycetemcomitans, is impaired. This damage in LAP neutrophils and macrophages is partially reverted via exogenous MaR1, phagocyte ingestion, intracellular ROS production, and clearance of the periodontal pathogens. These functions were also enhanced in phagocytes from healthy patients via MaR1.

Bacterial clearance is critical for controlling infection and its resolution. Previous studies have proven that MaR1 enhances the phagocytosis of both the organisms in the concentration identified from endogenous production. The addition of exogenous MaR1 has enhanced the clearance ability of LAP macrophages and normalized the neutrophil function.

Following phagocytosis, intracellular reactive oxygen species generation is crucial for oxidative bacterial killing within the phagocytes. MaR1 has been shown to increase the ROS levels in the phagocytes, enhancing phagocytosis [19].

Maresins and their role in periodontal regeneration

Restoration of periodontal tissue after periodontal inflammation and destruction is a very complex process that involves the synergistic action of various cells and growth factors. The tooth sits firmly in the socket and is connected to the alveolar bone by the periodontal ligament. The stem cells of the periodontal ligament (PDLSCS) play a major role in the tooth-bone attachment, regeneration, and maintenance of periodontal tissue homeostasis and masticatory function. The periodontium harbors a continuous presence of microorganisms that induce a constant inflammatory response. Therefore, the function of PDLSC is controlled by the proinflammatory and proresolution mediators of inflammation.

Periostin plays a vital role in periodontal tissue development. It promotes the fibrillogenesis of collagen and the migration of osteoblasts, fibroblasts, and stem cells [20]. Mediators of inflammation like TNF-a downregulate periostin in PDLSCs and periodontal ligament cells. Tenomodulin is a maturation marker for periodontal ligament cells of erupted teeth where it enhances the maintenance or maturation of PDL by upregulating cell adhesion. Tenomodulin-expressing stem cells have an enhanced ability to induce teno/ligamentogenesis, which is necessary for tissue regeneration when continually under tensile-occlusive stress. a-Smooth muscle actin (a-SMA) is another marker that is critical for self-renewal of the tissue. Along with tenomodulin, a-SMA improves stem cell contractility. Maresin upregulates periostin, tenomodulin, and a-SMA at protein and gene levels, strongly suggesting that the impact of proinflammation is reversed by the proresolution phase of inflammation and the PDL-like cell phenotype of the hPDLSCs is restored [20].

Regulation of autophagy by maresins

Autophagy, a cellular degradation pathway by lysosomes, plays a critical role in the mechanism of inflammatory diseases such as periodontitis [21]. Autophagy has numerous functions that have been confirmed to have anti-inflammatory properties. It is well known now that autophagy has a central role to play in inflammation and immunity. Anything affecting autophagy may affect inflammation. Studies have revealed that maresin-1 plays a role in cell inflammation and autophagy [22,23]. Also, studies have shown us that autophagy is mediated by the activation of the GSK-3ß pathway [24], and a major downstream target of GSK-3ß is ß-catenin. A study by Li et al. revealed that MaR-1 plays an important role in PDL cell survival and inflammation by regulating cell autophagy and activating the GSK-3ß/ß-catenin signal pathway [25]. MaR1 treatment activated the GSK-3ß pathway (which upregulated autophagy) and was also able to inhibit ß-catenin expression. The study suggested that MaR-1 is involved in the survival of cells by mediating the autophagy pathway and producing proinflammatory cytokines. Considering these results, there may be new perspectives on how to treat periodontitis in humans.

Maresins and their role in promoting wound healing

Macrophages are key players in resolving any inflammation and also promote wound healing and tissue regeneration. Given that M2 macrophages produce maresins, it is likely that maresins are key mediators between the resolution of inflammation and the stages of wound healing and regeneration. It was also discovered that MaR1 assists in the healing of vascular injury [26] and was organ protective in a lung inflammation model in mice [16]. An animal study was conducted on the role of topical application of maresins in extraction socket healing and socket regeneration. It was found that MaR1 hastened wound healing, promoted bone fill in the socket, preserved the alveolar ridge, and reduced postoperative pain [27]. Some researchers observed that MaR1 reduced the proinflammatory cytokines and reactive oxygen species, reducing skin edema in an animal model [28]. Another study stated that TNF-α inhibits keratinocyte proliferation [27]. MaR1 is known to inhibit the production of TNF-α [29]. Hence, considering all these possible factors, the potential mechanism for wound healing by maresins seems to be indirectly through the mechanism of resolution of inflammation. The mechanism by which maresins cause bone fill by directly acting on the osteoblasts or indirectly through the resolution of inflammation is yet to be discovered.

## Conclusions

Inflammation is a protective response to eliminate pathogens and promote healing; however, an exaggerated inflammatory response can be pathological and can cause irreversible tissue damage. In periodontitis, bone loss is a consequence of an exaggerated inflammatory response. It is therefore important to dampen the inflammation in time before it progresses to bone loss. The mediators of inflammation, i.e., lipoxin, protectins, resolvins, and maresins, are lipids derived from omega-6 or omega-3 fatty acids. Maresins are macrophage mediators that act on specific receptors, i.e., RORα and LGR6, and perform various functions. They promote phagocytosis of M2 macrophages, inhibit infiltration of neutrophils, and stimulate Treg cell formation. They also inhibit the expression of proinflammatory cytokines. Studies have shown that maresins have a positive role in the treatment of aggressive periodontitis. They promote periodontal regeneration, autophagy, and wound healing. Thus, maresins are instrumental in curbing inflammation and helping the tissues heal. Presently, animal studies are ongoing, and future clinical trials are required before they can be used in the management of periodontitis.
